# Preliminary outcomes on phenotypic and genetic parameter estimates for body weight of indigenous Tswana goats in Botswana

**DOI:** 10.1007/s11250-024-04144-2

**Published:** 2024-10-09

**Authors:** J. Yiga-Kibuuka, K. Raphaka, P. I. Monau, S. J. Nsoso

**Affiliations:** 1 P. O. Box 47412, Gaborone, Botswana; 2 National Agricultural Research and Development Institute, Private Bag 00177, Gaborone, Botswana; 3 Faculty of Animal and Veterinary Sciences, Department Animal Sciences, Botswana University of Agriculture and Natural Resources, Private Bag 0027, Gaborone, Botswana

**Keywords:** Average daily gain, Genetic correlations, Heritability, Tswana goat

## Abstract

The aims of this study were to estimate the genetic and phenotypic parameters for growth traits and evaluate genetic trends on 585 indigenous Tswana goats. The population was maintained under low input production system at the Department of Agricultural Research in Lesego ranch, Botswana, from 2005 to 2008. Data included birth weight (BW), weaning weight (WW), weight at 8 months (PW), yearling weight (YW), pre-weaning average daily gain (ADG) and two post weaning average daily gains (ADG2 and ADGYW). Data was analysed using general linear model of SAS to determine non-genetic effects. Estimation of genetic and phenotypic parameters were estimated using ASREML fitting an animal model that accounted for fixed effect of parity, sex, type of birth and year of birth. Least squares means for BW, WW, PW and YW were 2.88 ± 0.03, 12.15 ± 0.17, 16.52 ± 0.28 and 21.04 ± 0.32Kg, respectively, while those for ADG, ADG2 and ADGYW were 74.52 ± 1.41, 28.78 ± 1.55 and 33.66 ± 2.28 g/day, respectively. Estimates of heritability for BW, WW, PW and YW were 0.79 ± 0.11, 0.63 ± 0.14, 0.32 ± 0.13 and 0.48 ± 0.16, respectively. The genetic correlations for all the traits studied were positive and moderate to high (0.48 to 0.82) whilst phenotypic correlations ranged from 0.21 to 0.72. Positive average genetic trends of 12.32% (WW), 13.39% (PW) and 7.38% (YW) were attained. The results have demonstrated the potential of this breed to be improved through selection.

## Introduction

The Tswana goat is an indigenous goat breed in Botswana mostly kept by resource-poor farmers as a source of food and revenue (Monau et al. 2017). It is found in all agro-ecological regions of the country under low input production environment. This breed retains superior thermoregulation capacity and has the ability to exploit low quality forage and water shortage (Katongole et al. 1996; Nsoso et al. 2004). It has shown to be less susceptible to local diseases such as heart water and other parasitic diseases (Nsoso et al. 2004). Lately, goats in general and this breed in particular has become even more important due to the ability to cope with climate challenges such as frequent incidences of drought and fluctuation in temperatures (Dzama 2016). However, this breed has never been genetically improved and is perceived to have relatively poor growth potential. In the past, indigenous breeds were replaced and crossed with exotic breeds, but this was unsuccessful under low-input maintenance (APRU 1984).

Tswana goat has the potential to be developed through within-breeding selection programme as it will assist in improving goat productivity, food security and conservation of inherent traits. A breeding program directed at improving indigenous Tswana sheep and goats for the low input production system in Botswana was initiated by the Department of Agricultural Research using breeding values as a selection tool (APRU 2007). The objective of the improvement program was to increase weight traits such as body weight and average daily gains of the animals while retaining the unique attributes mentioned above. Estimates of genetic parameters of body weight and average daily gains have been reported on different indigenous goat breeds with limited reports on genetic trends (Bosso et al. 2007; Hasan et al. 2014). To date, no genetic parameters for growth traits have been estimated for the Tswana goat. Therefore, the objectives of this study were to estimate genetic and phenotypic parameters for growth traits as well as genetic trends of the indigenous Tswana goats kept under semi-intensive system from 2005 to 2008.

## Materials and methods

### Location and flock management

The records for this study were obtained from Lesego Ranch in the central district of Botswana. This ranch was established by the government of Botswana in 1990 for selection, breeding, and multiplication of indigenous Tswana goats and for distribution to local farmers As well as for research purposes. The animals are a representative of the Tswana breed as they were initially purchased from all different agro-ecological regions of Botswana to make sure that the inherent variation is captured. The grass species on the ranch include *Panicumspp*, *Eragrostis superba* and *Bracharianego pedata* while the browse tree species include *Colophospermum mopane*,* Combretum* and Acacia species. Management of the flock has been previously described in APRU (2006). Briefly, the animals were managed semi-intensively that is having access to unimproved pastures and mineral lick (Di-Calcium Phosphate-salt mixture). Animals had unrestricted access to water and all the necessary vaccination routines and anthelmintic treatments were undertaken.

Does were mated in single sire kraals in August of every year with a ratio of 1 buck to about 15-20-does. Maiden does were first mated at about 18–20 months of age and stayed in the flock until culling or death. All bucks were first used at about 24 months of age for single breeding season. Kidding occurred once a year around February/Mach during summer. The breeding strategy was performed to maintain inherent genetic variability. All kids were identified with plastic ear tags and weighed within 24 h of birth. Kids were weaned at an average age of 120 days to mirror communal management setup in Botswana. All animals were weighed on monthly intervals up to 12 months of age, then sold or kept for breeding.

### Data preparation and analysis

The data used in this study consisted of a total of 585 kids from 337 dams and 28 sires born between 2005 and 2008. Body weight was recorded at birth (BW), at weaning (WW) (adjusted to 120 days), at 8 months (PW) (adjusted to 240 days), and at 12months (YW). Average daily gains were computed; from birth to weaning (ADG), from weaning until 8-month (ADG2) and from 8 to 12 months of age (ADG3). Non-genetic effects included the parity od the does, sex, type of birth of kid (single of multiple) and year of birth. The means, standard deviations, coefficients of variation, minimum and maximum values for growth traits of the indigenous Tswana goats are given in Table 1.

The general linear model procedure of SAS (version 9.0) was used to select the main effects (sex, birth type, age of dam, and year of birth) and the two-way interactions that influenced the various body weights. Effects that significantly (*P* < 0.05) influenced the records were included in the model. Multiple comparisons were done using Duncan’s multiple range tests and the following model was used;

$${{\rm{Y}}_{{\rm{ijklm}}}}{\rm{ = \mu + }}{{\rm{A}}_{\rm{i}}}{\rm{ + }}{{\rm{B}}_{\rm{j}}}{\rm{ + }}{{\rm{X}}_{\rm{k}}}{\rm{ + }}{{\rm{Z}}_{\rm{l}}}{\rm{ + }}{{\rm{E}}_{{\rm{ijklm}}}}$$

where;

Y_ijklm_: = growth trait (BW, WW, PW, YW, ADG, ADG2 or ADG3),
µ: = population mean.
A_i_: = effect of A^th^ year of kidding (4 levels, from 2005 to 2008).
B_j_: = effect of B^th^ birth type (single or multiple)
X_k_: = effect of X^th^ sex of kid (male or female)
Z_l_: = effect of Z^th^ parity (. 1–2, 3–4, and 5 or above)
Eijklm: = random residual error.

Genetic parameters were estimated using ASReml2 (Gilmour et al. 2009) using the model.

$${\rm{Y = X\beta + Z}}a{\rm{ + \varepsilon }}$$

where,

Y is the vector of records.

β, ɑ, and ɛ are vectors of fixed, direct additive and residual effects, respectively.

X, Z is the incidence matrices.

Bivariate analysis using the same model was used for estimating the genetic and phenotypic correlations. The data structure did not permit the inclusion of maternal genetic effects in the model as there was no pedigree information on most of the dams. Means for estimated breeding values (EBVs) of the animals by year of birth were calculated to estimate genetic trends.

**Table 1:** Number of goats, means, standard deviations (SD), minimum and maximum values and coefficients of variation (cv%) for weights at different age groups of Tswana goats

Variable	Number	Mean	SD	Minimum	Maximum	CV(%)
BW(Kg)	585	2.88	0.63	1.00	5.50	21.98
WW(Kg)	448	12.15	3.61	4.01	23.55	29.70
PW(Kg)	336	16.52	5.13	7.13	30.51	31.08
YW(Kg)	276	21.04	5.30	11.67	39.11	25.18
ADG(g)	398	74.52	28.20	14.29	160.33	37.84
ADG2(g)	314	28.78	27.40	-32.00	122.95	95.20
ADG3(g)	256	33.66	36.44	-72.58	121.85	108.28

BW = Birth Weight; WW = Weaning Weight; PW = 8 Month Weight and YW = Yearling Weight

## Results

The least square means and standard errors of body weights are presented on Table 2. The analysis indicated that parity of does, sex, type of birth and year had significant effect (*P* < 0.05) on body weight at different ages. Kids born from younger does generally had lower weights. Throughout the study period, the male kids were significantly (*P* < 0.05) heavier than female kids at all ages. The single born kids outweighed their multiple born counterparts by 61 g at birth and continued to be significantly heavier in subsequent ages. There was significant variation of weight across the years; in 2005, WW, PW and YW were significantly lower compared to other years whilst birth weights were significantly lower in 2008 (Table 2).

The least squares mean for pre-weaning average daily gains (ADG) were higher than the post-weaning average daily gains (ADG2 and ADG3). Type of birth, year of birth as well as sex of kid had significant (*P* < 0.05) effects on average daily gains. Male kids had higher average daily gains than females at all stages of growth. Single born kids only had a distinct advantage in weight gain over those born in multiple births before weaning though at later stages there was no significant difference between them. Marked year-wise differences (*P* < 0.05) for all growth traits of kids were observed but with no definite pattern among different years of study (Table 2).

**Table 2:** Least squares means and standard errors (± SE) for birth, weaning, post-weaning and yearling weights for indigenous Tswana goats

	*N*	BW ± SE (kg)	WW ± SE (kg)	PW ± SE (kg)	YW ± SE (kg)	ADG(g)	ADG2(g)	ADG3 (g)
Overall	585	2.88 ± 0.03	12.15 ± 0.17	16.52 ± 0.28	21.04 ± 0.32	74.52 ± 1.41	28.78 ± 1.55	33.66 ± 2.28
Parity of does		***	ns	ns	*	ns	ns	ns
1	103	2.73 ± 0.06^b^	12.50 ± 0.34^b^	17.47 ± 0.40^b^	21.45 ± 0.64	78.07 ± 2.97^b^	37.04 ± 2.65^b^	34.74 ± 4.83^b^
2	312	2.97 ± 0.04^b^	12.90 ± 0.22^a^	18.20 ± 0.30^a^	20.48 ± 0.49	82.28 ± 1.90^a^	39.49 ± 2.02^a^	41.71 ± 3.83^a^
3	170	3.08 ± 0.05^a^	13.00 ± 0.30^a^	18.16 ± 0.37^a^	21.59 ± 0.61	81.92 ± 2.48^a^	38.43 ± 2.50^a^	47.42 ± 4.58^a^
Sex		***	**	***	***	**	*	***
Female	286	2.84 ± 0.04^b^	12.29 ± 0.23^b^	16.99 ± 0.29^b^	20.82 ± 0.47^b^	76.75 ± 1.98^b^	35.36 ± 1.98^b^	32.49 ± 3.65^b^
Male	299	3.01 ± 0.04^a^	13.31 ± 0.24^a^	18.89 ± 0.30^a^	24.95 ± 0.50^a^	84.77 ± 2.00^a^	41.27 ± 2.08^a^	50.09 ± 3.84^a^
Type		***	***	***	***	***	ns	ns
1	184	3.23 ± 0.04^a^	14.83 ± 0.25^a^	20.17 ± 0.31^a^	25.02 ± 0.52^a^	95.10 ± 2.12^a^	39.02 ± 2.11	40.59 ± 3.90
2	402	2.62 ± 0.03^b^	10.77 ± 0.23^b^	14.77 ± 0.30^b^	20.75 ± 0.47^b^	66.41 ± 2.00^b^	37.62 ± 2.02	42.00 ± 3.71
Year	***	***	***	***	***	***	***	***
2005	285	3.06 ± 0.04^a^	11.60 ± 0.20^c^	12.87 ± 0.25^c^	18.99 ± 0.42^c^	70.17 ± 1.61^c^	7.49 ± 1.58^c^	49.80 ± 3.09^a^
2006	77	3.00 ± 0.07^a^	12.39 ± 0.59^b^	18.77 ± 0.82^b^	24.77 ± 1.36^a^	75.12 ± 4.99^b^	52.78 ± 5.66^a^	56.15 ± 10.89^a^
2007	112	2.92 ± 0.05^b^	14.64 ± 0.26^a^	20.85 ± 0.28^a^	22.68 ± 0.45^b^	97.71 ± 2.53^a^	49.10 ± 1.83^ab^	14.36 ± 3.25^b^
2008	112	2.73 ± 0.06^c^	12.57 ± 0.37^b^	19.28 ± 0.47^ab^	25.10 ± 0.71^a^	80.01 ± 3.04^b^	43.91 ± 3.36^b^	44.85 ± 5.40^a^

Means with the same letter are not significantly (*P* < 0.05) different. ns = not significant(*P* > 0.05). * *P* < 0.05; ** *P* < 0.001 ***. BW = Birth Weight; WW = Weaning Weight; PW = 8 Month Weight and YW = Yearling Weight

The estimates of heritability for body weights were high and decreased with age. The genetic correlation of body weights ranged from intermediate to high and were all positive. The traits with the highest estimated genetic correlation were between weaning weight and eight month weights (1.0) and between birth and weaning weight (0.82). The phenotypic correlations were positively low to high, ranging from 0.21 to 0.72 (Table 3).

**Table 3:** Estimates of heritability (diagonal), genetic (above diagonal) and phenotypic (below diagonal) correlations of body weights

Traits	BW(SE)	WW(SE)	PW(SE)	YW(SE)	σ^2^_*P*_(SE)
BW	**0.79(0.11)**	0.82(0.10)	0.78(0.16)	0.48(0.19)	0.35(0.03)
WW	0.32(0.05)	**0.63(0.14)**	1.03(0.05)	0.51(0.18)	8.64(0.70)
PW	0.21(0.06)	0.72(0.03)	**0.32(0.13)**	0.66(0.18)	9.57(0.78)
YW	0.21(0.07)	0.46(0.05)	0.55(0.05)	**0.48(0.16)**	20.11(1.89)

σ^2^_P_ = phenotypic variance, BW = Birth weight; WW = Weaning weight; PW = 8 Month weight and YW = Yearling weight

**Fig. 1:**
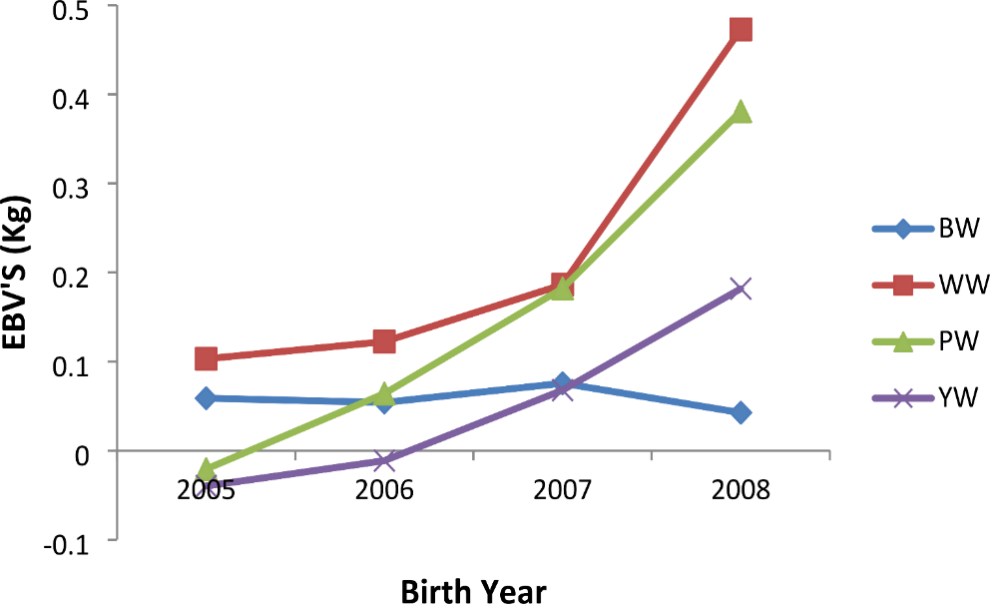
Trend of Estimated breeding values by year of birth for birth weight (BW), weaning weight (WW), post weaning (PW) and yearling weight (YW) in indigenous Tswana goats

The mean estimated breeding values (EBV) for WW, PW and YW in indigenous Tswana kids showed an increasing trend for the four years of the breeding program while for birth weight it remained steady and dropped slightly in some years. On average, the EBV’s for WW, PW and YW increased by 1.75%, 2.58% and 2.68% whilst the EBV’s for BW reduced by 3.97% per year throughout the years of the study (Fig. 1).

## Discussion

Body weights and average daily gains are important economic growth traits for improving production performance for selective breeding. The average birth, weaning and yearling weights observed in this study are lower than those reported previously on the same breed (Madibela et al. 2002; APRU 2006). However, similar findings were reported in Tanzanian blended goats (Das et al. 1996), common African goats (Mourad and Anous 1998) and West African goats (Bosso et al. 2007). The lower weights could be attributed to the poor source of feed during the years of data collection. The observed effects of sex on body weights could be due to sex hormone effects. Baneh and Hafezian (2009) stated that oestrogen hormone has a limited effect for growth in female’s hence they have smaller bodies than males. The results are in agreement with Al-Saef (2013) and Hasan et al. (2014).

The effect of parity on body weights recognised in this study is consistent with Boujenane and El Hazzab (2008) but different from Al-Shorepy et al. (2002). The increase in parity with age is associated with better development of reproductive organs (such as the uterus) and better nutrition to the foetus (Browning et al. 2004). The younger does are not at their mature weight, hence partitioning of nutrients to complement their growth and foetus growth. Furthermore, the advantage of single kids over multiple births can be linked to less competition to nutritional quantity during gestation period and milk supply from their does. Similar phenomena have been reported by Boujenane and El Hazzab (2008), Sodiq (2012) and Al-Saef (2013).

The observed variation of body weights over the years of study could be due to different sample sizes, climatic changes, availability of feed and body conditions of the does. The pre-weaning growth rate of 74 g per day observed in this study was similar to the findings of Das et al. (1996) and Thiruvenkadan et al. (2009) but lower than 91.5 g from previous research of Tswana goats (Madibela et al. 2002). Van Niekerk and Casey (1996) reported average pre-weaning daily gains of 160 g on Boer goats. The low average daily gains observed could reflect low genetic potential for growth and milk production on Tswana goats.

The estimate for direct heritability for birth weight in this study is in accordance with the report by Mourad and Anous (1998) in common African and Alpine crosses goats but greater than those reported by Bosso et al. (2007) in West African dwarf goats (0.50), Boujenane and El Hazzab (2008) in Draa goats (0.39) and Zhang et al. (2008) in Boer goats (0.36).The estimate of heritability for weaning weight (WW) was higher than the estimates reported in the literature which ranged from 0.21 to 0.35 in different goat breeds (Al-Shorepy et al. 2002; Al-Saef 2013; Hasan et al., 2014). The variation could be due to breed, sample sizes and environmental factors. In this study, the maternal effects could have confounded with the direct variance leading to significantly higher pre-weaning values. Zhang et al. (2009) stated that models which did not include maternal effects tend to overestimate direct heritability for birth weight and weaning weight. Potential response to selection based on these values would also be overestimated.

The estimates of post weaning heritability recognised in this study were within the range reported by Bosso et al. (2007) and Hasan et al. (2014) but greater than 0.11–0.12 reported on Jamunapari goats (Rout et al. 2018). The estimates for yearling weight were lower than those reported by Bosso et al. (2007) and Hasan et al. (2014) on West African Dwarf goats and Ettawa goats, repectively. Lower heritability estimates have been reported in different goat breeds ranging from 0.07 to 0.11 (Zhou et al. 2002; Mugambi et al. 2007; Thiruvenkadan et al. 2009; Gowane et al. 2011). The differences could be due to breed type, sampling errors, environment and management. Nonetheless, the results imply that selection gains for growth will be achieved at eight and twelve months.

The estimates of additive genetic correlations among the traits in this study were high and positive indicating good genetic association among the traits and relevance for selection programmes. The genetic association between birth weight (BW) and weaning weight (WW) were higher than the values obtained by van Niekerk et al. (1996) in Boer goats but in agreement with Bosso et al. (2007) and Zhang et al. (2009) on West African dwarf goats and Boer goats, respectively. In addition, the observed phenotypic correlations among traits conformed to those reported by Maghsoudi et al. (2009) and Wang et al. (2011). These estimates reflect on the effects of environmental factors on early growth traits of Tswana goats. The genetic and phenotypic correlations between traits are essential in predicting indirect responses to selection, determining the optimum weight and possible response to selection in a multi-trait selection program.

The positive increasing genetic trends observed in this study were in accordance with Bosso et al. (2007) and Rout et al. (2018). The results show the rate of improvement and selection effectiveness on Tswana goat. The high genetic trend at weaning indicates the opportunity for increased genetic progress when selecting animals at an earlier age of growth hence the ability to make selection decision earlier in the animal life. This is supported by the positive genetic correlations between weaning weight and other growth parameters. Nonetheless, data should be collected over more years to get the accurate breeding values of weight on Tswana goats. Type of birth, sex and parity were the major factors affecting growth rate in Tswana goats. The heritability estimates indicated that selection for weight should be performed on the post-weaning traits preferably at eight-months. A positive response could be expected in other traits owing to the high and positive correlations. Farmers should be capacitated with management of animals and record keeping. Since maternal effects could play a significant role in the expression of pre-weaning traits, it is important to know the extent of both maternal and environmental effects in addition to the direct additive component for optimum breeding programmes and selection efficiency. Although the data might have been collected in the past fifteen years, this paper represents efforts to characterise indigenous animal genetic resources and is an important first step at national and regional level. This the only very crucial available data for this indigenous animal and will be used as baseline data for future research.

## Data Availability

The data used on this study is available at the Botswana Ministry of Agricultural Development and Food Security, Department of Agricultural Research. The data maybe provided on request through the corresponding author.
